# Can High b Value Diffusion Be a Surrogate Marker for PET—A MR/PET Study in Neurooncology Set Up

**DOI:** 10.3389/fneur.2021.627247

**Published:** 2021-09-24

**Authors:** Sandhya Mangalore, Sriharish Vankayalapati, Shumyla Jabeen, Arun Kumar Gupta, Pardeep Kumar

**Affiliations:** Department of Neuroimaging and Interventional Radiology, National Institute of Mental Health and Neurosciences, Bangalore, India

**Keywords:** PET-MR, CNS tumors, FDG-PET, high value diffusion weighted imaging, glioma

## Abstract

**Purpose:** Hybrid whole-body magnetic resonance/positron emission tomography (MR/PET) systems are new diagnostic tools enabling the simultaneous acquisition of morphologic and multiparametric functional data, which allow for a diversified characterization of oncological diseases. This study aimed to compare the diagnostic ability of MRI with the diffusion-weighted image (DWI), and simultaneous integrated positron emission tomography MR/PET to detect malignant lesions and elucidate the utility and limitations of these imaging modalities in preoperative and postoperative follow up in cancer patients.

**Material and Methods:** A total of 45 patients undergoing simultaneous MR/PET for CNS ICSOL in our institution between January 2016 and July 2020 were considered in this study. Post-processing was done in Siemens syngo software to generate a b2000 image. This image was then inverted to grayscale and compared with the NAC image of PET.

**Results:** The lesion-based sensitivity, specificity, positive predictive value, and negative predictive value for DWI were 92.3, 83.3, 97.3, and 62.5%, respectively (at 95% CI and *p* was 0.000). The lesion-based sensitivity, specificity, positive predictive value, and negative predictive value for PET were 97.4, 71.4, 94.9, and 83.3%, respectively (at 95% CI and *p* was 0.000). The lesion-based sensitivity and specificity of DWI were comparable with those of PET.

**Conclusions:** Although DWI and FDG-PET reflect different tissue properties, there is an association between the measures of both methods in CNS tumors probably because of the coupling of cellularity with tumor metabolism as seen on FDG and other PET tracers. Our study shows that DWI acts as a surrogate biomarker for FDG PET and other tracers in tumors. The method of DWI image generation is simple, radiation-free, and cost-effective in a clinical setup. The simultaneous DWI-PET study provides evidence and confirms the role of DWI in surveillance imaging of tumors.

## Introduction

The hybrid whole-body magnetic resonance positron emission tomography (MRPET) system is a new diagnostic tool. It enables the simultaneous acquisition of morphologic and multiple functional data and thus a diversified characterization of oncological diseases (1, 2).

Our study focuses on the use of simultaneous MR/PET. The temporal correlation of MRI and PET in a single sitting is possible with MR/PET and various MRI parameters and PET tracers can be compared with high spatial and temporal resolution. MRI is a multiparametric imaging technique and when combined with simultaneous PET is better than PET computed tomography.

Many MR/PET studies correlate with multiple advanced imaging parameters like diffusion-weighted imaging (DWI) and Perfusion weighted imaging (PWI) studies on the grading of gliomas, enabling the prediction of recurrence. It is possible to differentiate recurrence vs. radiation necrosis and the false negative in each modality is known (3–6). PET also has many tracers, each with its specificity and sensitivity (7–10).

Each modality has inherent limitations, for instance, false-positive uptake of FDG PET is due to the immediate effects of cyberknife and gamma knife, and post-radiotherapy related inflammatory changes (11). The resolution of PET at best is 4–6 mm (12). Many small lesions in the brain may be missed at this resolution in a post-operative case. MRI parameters on the other hand, such as PWI, MRS, contrast enhancement patterns have specificity and sensitivity and since these are ROI-based measurements, inherent flaws exist (12, 13). MRS and PWI can help grade tumors but since they use large voxel sizes, they have low specificity (14). Contrast enhancement pattern, which represents blood-brain barrier imaging, helps to correlate, but pseudoprogression and pseudo regression are known entities (15).

Though many DWI techniques have been discussed, no study had access to simultaneous MR/PET, especially in a neuro-oncology setup. However, the clinical benefits of simultaneous MR/PET imaging need to be balanced against the relatively high cost and availability of such an approach.

This paper discusses DWI as a potential MRI biomarker for FDG PET and other tracers. Moreover, this marker is simple, radiation-free, and cost-effective, making clinical translations more straightforward. This study compares DWI with PET images and aims to establish the sensitivity specificity and pitfalls in neurooncology.

We hypothesize that DWI and PET will have a good correlation as both show signal changes and uptake is based on cellularity. Though this paper mainly focuses on the role of MR/PET in cases of glioblastoma, we also briefly describe our experience with other histotypes of CNS tumors and also with other PET tracers as well.

## Materials and Methods

Approval was obtained from our institutional ethics committee. Patient consent was waived for this retrospective observational analysis of anonymized data. The study considered all patients with intra-cranial space-occupying-lesions (ICSOL) (all grades of gliomas and various other histotypes) evaluated with MR/PET in our institution between January 2016 and July 2020. A total of 45 cases with simultaneous MR/PET imaging are included in the study. All patients had histologically proven malignancies or suspected malignancies. Whole-brain PET images along with multiplanar and multi-sequence MR images were acquired in 3-D mode after i.v. injection of 18F-FDG using a simultaneous Siemens mMR Biograph PET/MR scanner. In a few cases different tracers like C-11methionine, F-18 choline was used as part of a clinical protocol.

This study aimed to compare the diagnostic ability of MRI with a diffusion-weighted image (DWI), and simultaneous integrated positron emission tomography MR/PET to detect malignant lesions and elucidate the utility and limitations of these imaging modalities in preoperative and postoperative follow up in cancer patients.

### DWI Image Acquisition

A routine multiparametric MRI was conducted including T2, SWI, and fluid-attenuated inversion recovery (FLAIR) sequences, axial and 3D T1-weighted, and GdT1w-MRI.

DWI was acquired in the axial plane before injection of contrast material for all patients by fast echo-planar T2^*^-weighted gradient echo sequence and used for generating ADC maps. The sequence parameters were: b-values 0 and 1,000 s/mm^2^,TR = 3,600 ms, TE = 81 ms, FOV = 23 cm, matrix = 128 × 128, voxel size-0.9 × 0.9 × 4 mm, 27 slices of 4 mm thickness 4 mm without spacing, NEX- 1.

### Image Interpretation

Two radiologists interpreted the MR/PET images in a blinded fashion. The lesion-specific signal intensity on DWI and ADC is compared with metabolism on PET images. Histopathology, all imaging findings, and follow-up scans served as a standard of reference. The DWI images were post-processed using Siemens Heathineers syngo software (version 05.01.0000.0061). The MR basics module in the software package has options for calculating the high b value of trace images and hence we generated images at b2000 to suppress the T2 shine-through the effects of gliosis and edema and also at high value there was good background suppression and increased conspicuity of recurrent foci. For ease of visual correlation, in this study, we aimed to obtain a PET-like image from DWI by inverting the b2000 trace image to grayscale, which has the potential to be an excellent biomarker in the neuro-oncology workup.

### Statistical Analysis

Data were collated offline on Microsoft Excel version 15.0.4 and statistical analysis was conducted with an online interactive statistics page using a 2 × 2 consistency table. Lesion-based sensitivity, specificity, negative predictive value, positive predictive value were calculated for DWI and PET modalities. The Yates corrected *P*-value < 0.005 was considered significant.

## Results

Anonymized MR/PET imaging data obtained in 45 patients with ICSOL were also used in the analysis and examined glioma of all grades (*n* = 35) and other histotypes (*n* = 10, including ependymoma, craniopharyngioma, meningioma, lymphoma, atypical rhabdoid tumor) with FDG tracer (*n* = 31), and other PET tracers such as C-11methionine (*n* = 8) and F-18 choline (*n* = 3).

Lesion-based sensitivity, specificity, positive predictive value and negative predictive value for DWI were 92.3, 83.3, 97.3, and 62.5% respectively (at 95% CI and *p* < 0.05). The lesion-based sensitivity, specificity, positive predictive value, and negative predictive value for PET were 97.4, 71.4, 94.9, and 83.3% respectively (at 95% CI and *p* is < 0.05). These lesion-based sensitivity, specificity of DWI were comparable with those of PET. The DWI showed significantly higher specificity than PET.

The salient observations noticed on visual analysis will also be discussed underneath as:

a) our experience with Glioma: high b value diffusion as a surrogate marker for FDG PET in the diagnosis of recurrence in brain tumors (Table 1).
b) Our experience with CNS tumors of other histotypes including the whole body work up (Table 2).
c) Our experience with other tracers (Table 2).

**Table 1:** Brief summary of GLIOMA cases evaluated with MRI FDG-PET.

	**MRI**	**Histopathology**	**DWI 2000**	**ADC 2000**	**PET**	**NAC vs. DWI**	**Other imaging findings**
1	Recurrence	Anaplastic oligodendroglioma, nos - who grade iii; right fronto insular	Bright	Dark	Hyper metabolism	Match	Linear and nodular enhancement and elevated perfusion
2	Recurrence in background of radiation necrosis	Recurrent glioblastoma who grade -iv; molecular information:idh-1(r132h) - positive, atrx - loss of expression, p53 – positive mib-1 labeling is high (25–30%) at foci	Bright	Dark	Hyper metabolism	Match	Swiss cheese enhancement and elevated perfusion
3	Recurrence	Anaplastic oligodendroglioma, who grade iii; left frontal.	Bright	Dark	Hyper metabolism	Match	Homogenous enhancement with elevated perfusion
4	Radiation necrosis		Bright	Dark	Hypo metabolism		Homogenous enhancement
5	Radiation necrosis		Iso	Iso	Hypometabolism	Match	Swiss cheese enhancement with no elevated perfusion
6	Recurrence	Anaplastic oligodendroglioma - who grade-iii - right frontal.	Bright	Dark	Hyper metabolism	Match	Swiss cheese with nodular foci on enhancement. Volume underestimation on PET
7	Recurrence	Glioblastoma [idh-1 (r132h) mutant] - who grade iv; right frontal.	Bright	Dark	Hypermetabolism	Match	Nodular foci of enhancement with elevated perfusion
8	Recurrence	Glioblastoma, who grade-iv, left parietal.	Bright	Dark	Hyper metabolism	Match	Swiss cheese with nodular foci on enhancement and elevated perfusion
9	Recurrence		Bright	Dark	Few areas of Hyper metabolism	Match	Heterogeneous enhancement
10	Radiation necrosis	Anaplastic astrocytoma -who grade iii; corpus callosum	Iso	Iso	Hypometabolism	Match.	No foci of enhancement or elevated perfusion
11	Recurrence in background of radiation necrosis	Infiltrating anaplastic astrocytoma, left occipital.	Bright	Dark	Hypermetabolism	Match. Lesion is more evident on DWI	Swiss cheese with thick areas of enhancement and elevated perfusion. Overestimation on pet.
12	Radiation necrosis		Iso	Iso	Hypometabolism	Match	No foci of enhancement
13	Radiation necrosis	Anaplastic mixed oligo-astrocytoma, who grade-iii.	Iso	Iso	Hypometabolism	Match.	Heterogeneous enhancement along the resection cavity
14	Recurrence		Bright	Dark	Hypermetabolism	Match	Very thick nodular enhancement.
15	Recurrence		Bright	Dark	Hypermetabolism	Match	Extensive parenchymal enhancement with elevated perfusion
16	Recurrence	Glioblastoma (epithelioid variant) who grade iv; left frontal	Bright	Dark	Hypermetabolism	Match	Thick parenchymal enhancement. Overestimation on pet
17	Recurrence	Radiation necrosis with foci of glial neoplasm consistent with anaplastic glioma [idh 1 (r132h) negative, atrx retained expression]; left frontal.	Bright	Dark	Hypermetabolism	Match	Swiss cheese kind of appearance with overestimation on ASL
18	Recurrence	Glioblastoma, idh mutant, who grade iv, left frontal (recurrent).	Bright	Dark	Hypermetabolism	Match	Near homogenous enhancement with elevated perfusion on ASL
19	Recurrence	Glioblastoma who grade iv	Bright	Dark	Hypermetabolism	Match	Heterogeneous enhancement with elevated perfusion
20	Recurrence	Anaplastic oligodendroglioma, who grade iii; nos, right parieto occipital	Bright	Dark	Hyper metabolism	Match	Swiss cheese with nodular enhancement
21	Recurrence with metastasis in body	High grade glioma suggestive of pilocytic astrocytoma with malignant transformation (Dedifferentiation)	Bright	dark	Hypermetabolism	Match	Metastatic deposits in the occipital lobe; posterior fossa and left paravertebral region.
23	Recurrence	Anaplastic oligodendroglioma grade iii,	Bright	Dark	Hypermetabolism	Match	Swiss cheese enhancement with nodular foci of enhancement with elevated perfusion.
24	Recurrence	Anaplastic oligodendroglioma who grade iii	Bright	Dark	Hypermetabolism	Match	Nodular focus of entertainment with elevated perfusion along margins
25	Recurrence in background of radiation necrosis		Bright	Dark	Hypermetabolism	Match	Areas of near heterogenous enhancement with elevated perfusion
	**MRI**	**Histopathology**	**ADC 2000**	**ADC values mean**	**SUV max**	**PET**	**Other imaging findings**
1	Recurrence	Anaplastic oligodendroglioma, nos - who grade iii; right fronto insular	Dark	667.58	5.8	Hyper metabolism	Linear and nodular enhancement and elevated perfusion
2	Recurrence in background of radiation necrosis	Recurrent glioblastoma who grade -iv; molecular information:idh-1 (r132h) - positive, atrx - loss of expression, p53 – positive mib-1 labeling is high (25–30%) at foci	Dark	825.3	8.2	Hyper metabolism	Swiss cheese enhancement and elevated perfusion
3	Recurrence	Anaplastic oligodendroglioma, who grade iii; left frontal.	Dark	906.6	21.9	Hyper metabolism	Homogenous enhancement with elevated perfusion
4	Radiation necrosis		Dark			Hypo metabolism	Homogenous enhancement
5	Radiation necrosis		Iso	1,636	7.5	Hypometabolism	Swiss cheese enhancement with no elevated perfusion
6	Recurrence	Anaplastic oligodendroglioma - who grade-iii - right frontal.	Dark	420	5	Hyper metabolism	Swiss cheese with nodular foci on enhancement. Volume underestimation on PET
7	Recurrence	Glioblastoma [idh-1 (r132h) mutant] - who grade iv; right frontal.	Dark	725.5	27	Hypermetabolism	Nodular foci of enhancement with elevated perfusion
8	Recurrence	Glioblastoma, who grade-iv, left parietal.	Dark	882	6.8	Hyper metabolism	Swiss cheese with nodular foci on enhancement and elevated perfusion
9	Recurrence		Dark	659.2	7.1	Few areas of Hyper metabolism	Heterogeneous enhancement
10	Radiation necrosis	Anaplastic astrocytoma -who grade iii; corpus callosum	iso	1,201		Hypometabolism	No foci of enhancement or elevated perfusion
11	Recurrence in background of radiation necrosis	Infiltrating anaplastic astrocytoma, left occipital.	Dark	831.7	11.9	Hypermetabolism	Swiss cheese with thick areas of enhancement and elevated perfusion. Overestimation on pet.
12	Radiation necrosis		Iso	818.8	5.9	Hypometabolism	No foci of enhancement
13	Radiation necrosis	Anaplastic mixed oligo-astrocytoma, who grade-iii.	Iso	1,340	10.6	Hypometabolism	Heterogeneous enhancement along the resection cavity
14	Recurrence		Dark	489		Hypermetabolism	Very thick nodular enhancement.
15	Recurrence		Dark	906	11.5	Hypermetabolism	Extensive parenchymal enhancement with elevated perfusion
16	Recurrence	Glioblastoma (epithelioid variant) who grade iv; left frontal	Dark	668	11	Hypermetabolism	Thick parenchymal enhancement. Overestimation on pet
17	Recurrence	Radiation necrosis with foci of glial neoplasm consistent with anaplastic glioma [idh 1 (r132h) negative, atrx retained expression]; left frontal.	Dark	670	14.1	Hypermetabolism	Swiss cheese kind of appearance with overestimation on ASL
18	Recurrence	Glioblastoma, IDH mutant, who grade iv, left frontal (recurrent).	Dark	812.7	14.9	Hypermetabolism	Near homogenous enhancement with elevated perfusion on ASL
19	Recurrence	Glioblastoma who grade iv,	Dark			Hypermetabolism	Heterogeneous enhancement with elevated perfusion
20	Recurrence	Anaplastic oligodendroglioma, who grade iii; nos, right parieto occipital	Dark	405	3.5	Hyper metabolism	Swiss cheese with nodular enhancement
21	Recurrence with metastasis in body	High grade glioma suggestive of pilocytic astrocytoma with malignant transformation (Dedifferentiation)	dark			Hypermetabolism	Metastatic deposits in the occipital lobe; posterior fossa and left paravertebral region.
22	Recurrence	GBM with metastasis	Bright	414	54.7	Hypermetabolism	Heterogeneous enhancement with elevated perfusion
23	Recurrence	Anaplastic oligodendroglioma grade iii	Dark	439	7.7	Hypermetabolism	Swiss cheese enhancement with nodular foci of enhancement with elevated perfusion.
24	Recurrence	Anaplastic oligodendroglioma who grade iii	Dark	816	8	Hypermetabolism	Nodular focus of entertainment with elevated perfusion along margins
25	Recurrence in background of radiation necrosis		Dark	998	111.4	Hypermetabolism	Areas of near heterogenous enhancement with elevated perfusion

**Table 2:** Brief summary of other CNS histotypes cases evaluated with MRI FDG-PET and other PET tracers.

**S.NO**	**Indication**	**Final diagnosis**	**DWI b-2000**	**ADC**	**PET**	**NAC vs. DWI**	**PET tracer**	**Comments**
1	Primary tumor	Anaplastic Ependymoma	Bright	Dark	Hypermetabolism	Match	FDG	
2	Primary CNS lesion	Demyelination- clippers	Bright	Dark	Hyper etabolism	Match	FDG	HP after a month was Tcell lymphoma likely due to low tissue volume for both DWI and PET scan.
3	Brain and whole body PET	Lymphoma	Bright	Dark	Hypermetabolism	Match	FDG	HP came as demyelination but DWI PET matched as lymphoma – biopsy site should be planned properly
4	Recurrence With SMART syndrome	Anaplastic mixed oligo astrocytoma (WHO Grade III); left thalamus	Bright	Dark	Hypermetabolism	Small focus recurrence match PET and DWIMismatch of large area of gyral uptake and normal DWI	FDG SPECT	Patient had sudden weakness and large uptake of FDG with DWI normal –SMART syndrome
5	Primary	Diffuse midline high grade glioma with thalamic extension	Bright	Dark	Hypermetabolism	Match	FDG	DWI PET showed high grade lesion. Surveillance imaging. DWI showed free diffusion likely due to post radiotherapy resolution.
6	Primary tumor	Pontine glioma	Center bright	Dark	Hypermetabolism	Match	FDG	
7	Recurrence	Case of Melanotic schwannoma with multiple intracranial and spinal dural deposits and also in the right pinna and left orbit.	iso	iso	Hypermetabolism	DWI is masked due to presence of susceptibility foci	FDG	In syndromes NF differentiating tumor undergoing high grade transformation is crucial possible with Whole body DWI imaging
8	Recurrence with metastasis in brain.	External auditory canal squamous cell carcinoma.	Bright	Dark	Hypermetabolism	Match	FDG	Near homogenous enhancement with intracranial extension to temporal lobe
9	Primary tumor	Lymphoma right external capsule	Bright	Dark	Hypermetabolism	Match	FDG	Remote hyper metabolism in bilateral frontal lobes mimicking multifocal lymphoma with no lesion noted on MRI.
10	Recurrence	k/c/o diffuse astrocytoma grade ii/iv	Bright	Dark	Hypermetabolism	Match	C11 METHIONINE	
11	Primary tumor	Low grade glioma oligodendroglioma	Subtle bright	Subtle dark	Subtle hyper metabolism	Match	C11 METHIONINE	
12	Post operative follow up scan.	No recurrence of glioma	iso	iso	Hypometabolism	Match	C11 METHIONINE	
13	Primary tumor	Gliosarcoma IDH wild type, who grade-iv- right parietal.	Bright	Dark	Hypermetabolism	Match	C 11 METHIONINE	
14	Pre surgical work up	Meningioma incidentally detected.	iso	iso	Hypermetabolism	Mismatch	C 11 METHIONINE	Methionine is taken up by low grade benign tumor such as meningioma. DWI appearance based on cellularity
15	Primary tumor	Diffuse midline glioma H3k27m mutant; who grade iv; left ventricle and thalamus	Bright	Dark	Hyper etabolism	Match	C11 METHIONINE	
16	Recurrence	Atypical teratoid rhabdoid tumor, who grade-iv, suprasellar.	Bright	Dark	Hypermetabolism	Match	C 11 METHIONINE	
17	Recurrence	Craniopharyngioma	iso	iso	Hypermetabolism	Mismatch	C 11 METHIONINE	Low grade tumors such as craniopharyngioma show c11 methionine uptake
18	Recurrence	Recurrence GBM	Bright	Dark	Hypermetabolism	Match	F18 CHOLINE	
19	GBM	GBM	Match	Match	Hypermetabolism	Match	F18 CHOLINE	
20	Primary tumor	Right parietal anaplastic clear cell Ependymoma. False negative PET is reported in clear cell variant of Ependymoma.	Bright	Dark	Hypometabolism	Mismatch	F 18 CHOLINE	Variable behavior on F18 choline PET is reported in clear cell variant

### Using MRPET in Glioma to Differentiate Recurrence From Radiation Necrosis

A total of 25 post-operative patients with brain tumors were studied with preoperative histopathology and FDG MR/PET between 2016 and 2020.

Out of 25, 10 were Grade III and 8 were Grade IV tumors. A detailed histopathology report of glioma was not available in seven cases, as they were referred cases.

Among the 10 cases with Grade III, there were four who had IDH mutation, four were of p53 type and five were positive for ATRX. Among the eight cases with Grade IV tumor, two had IDH mutation, three were p53 subtype and one had ATRX mutation. The grade was based on a mitotic index. Of Grade IV cases, seven were of GBM tissue subtype and one was pilocytic astrocytoma subtype. In Grade III, six cases were of Oligodendroglioma subtype, one was of mixed Oligo astrocytoma subtype, and thee of anaplastic astrocytoma subtype.

**Figure 1:**
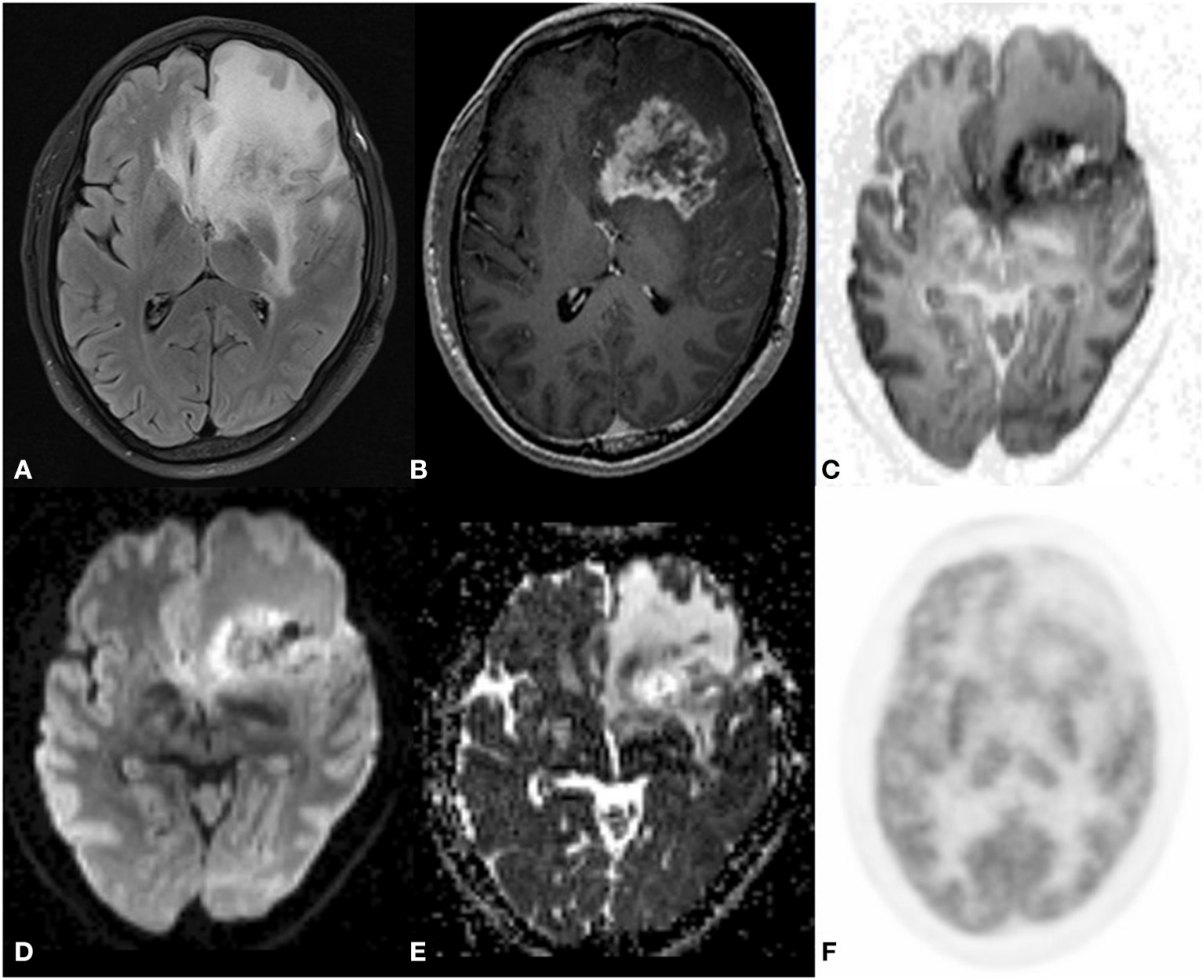
A 43 year old male postoperative case of Glioblastoma IDH mutant in the left frontal lobe, 5 year follow up MRI FDG PET axial images T2-FLAIR **(A)** post-contrast T1 **(B)** Inverted DWI **(C)** DWI b2000 **(D)** ADC **(E)** NAC PET **(F)** showed a T2 heterogeneous hypointense lesion with Swiss cheese and nodular enhancement and elevated perfusion. The lesion shows DWI restriction with hypermetabolism on PET. There is a match on the inverted DWI image and the NAC PET image. Suggestive of recurrence.

**Figure 2:**
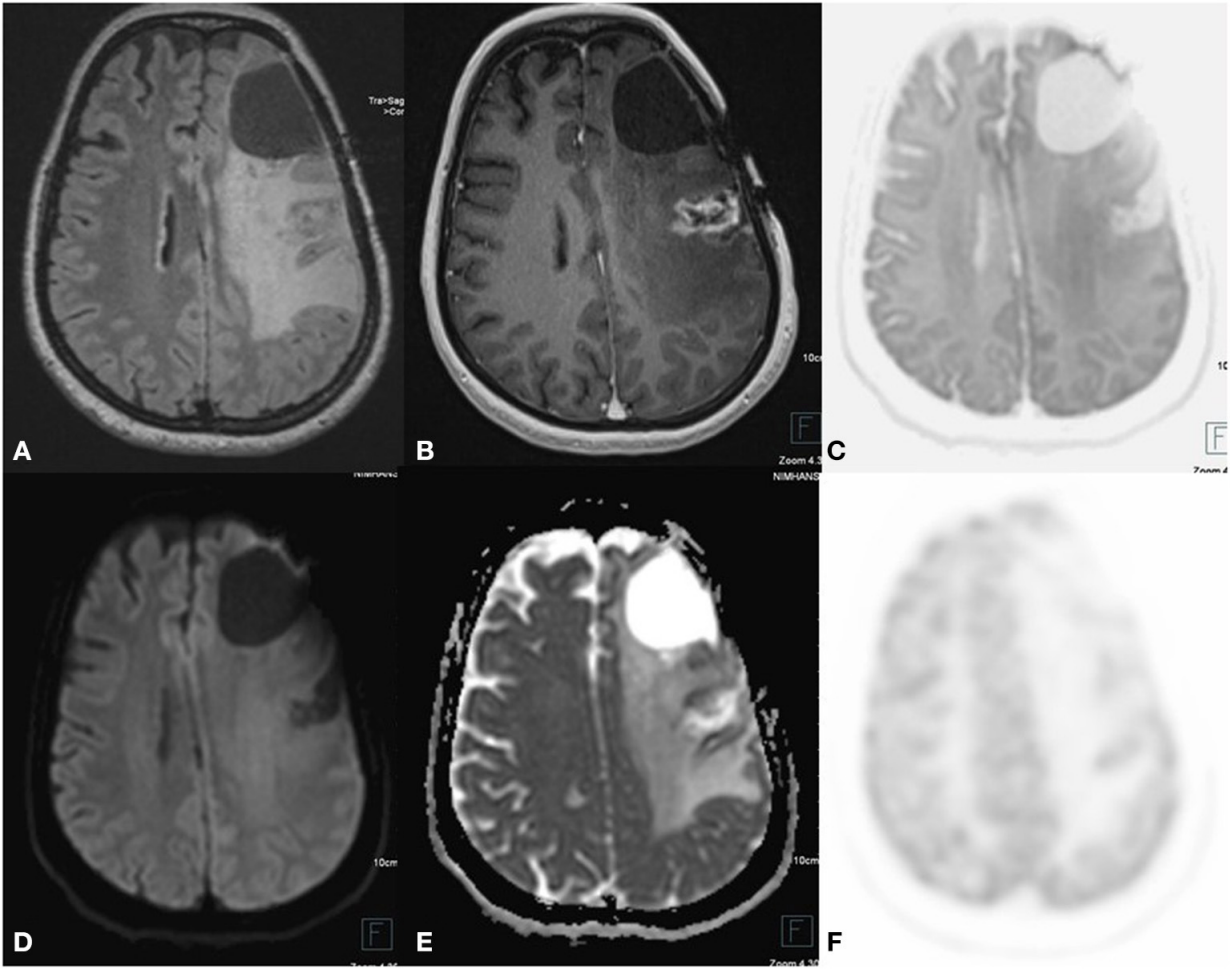
A 24 year old female postoperative, post-RT case of Glioma left frontal lobe, 3 year follow up MRI FDG PET axial images T2-FLAIR **(A)** post-contrast T1 **(B)** Inverted DWI **(C)** DWI b2000 **(D)** ADC **(E)** NAC PET **(F)** show a postoperative cavity in the left frontal lobe with Swiss cheese-like enhancing lesion posterior to postoperative either no elevated perfusion. Neither DWI restriction nor elevated metabolism on PET is seen, which is suggestive of radiation necrosis.

MR/PET was done in 25 cases of glioma and reported as recurrence (*n* = 20) (Figure 1) and radiation necrosis (*n* = 5) (Figure 2) based on imaging findings. On follow-up, histopathology was available in 16 cases in the recurrence group. Recurrence on PET was noted in 20 cases with radiation necrosis in five cases. In Grade IV, five had a recurrence and among Grade III, seven had a recurrence, and three had necrosis on PET. We used PET as a reference to standardize DWI in differentiating necrosis vs. recurrence. When B2000 DWI and PET findings were compared, very similar imaging findings were noted among both modalities concerning recurrence and the radiation necrosis group complementing each other.

On comparing PET -DWI for tumor volume, overestimation or underestimation of tumor volume was noted as compared to structural imaging. Since the intensity of PET is based on the uptake, tumors with high SUV can have a spillover effect with overestimation of tumor volumes in three cases and tumors with low SUV and below an absolute standard can appear smaller as noted in one case as compared to the DWI image. DWI is based on cellularity and FDG on metabolic uptake and hence this disparity in volume (1). In MRI of CNS tumors with the heterogeneous landscape, noting the volume of the tumor, cellularity on DWI, and ADC and SUV uptake measurements are important for prognosis and treatment response, which again highlights the need for multiparametric imaging.

On CEMRI, recurrence post-radiotherapy was considered when there was a nodular enhancement in the background of the Swiss cheese pattern of enhancement. On the correlation between DWI, PET, and CEMRI, false-positive recurrence was noted in two cases CEMRI but DWI and PET were suggestive of necrosis. Similarly, four cases showed false negative for recurrence on CE MRI when compared with DWI -PET.

PWI data was available in 22 cases. On post-processing ROI was placed on the enhancing portion on CEMRI.14 cases showed elevated perfusion values consistent with recurrence and the remaining eight cases did not show any elevated perfusion that was suggestive of necrosis. When the correlation between DWI, PET, and PWI was done, recurrence was missed in 4 cases i.e., false negative on PWI. This pitfall might be due to wrong ROI placement based on the standard enhancing portion on CEMRI rather than planning on either DWI or PET. Non-enhancing recurrence foci can be missed by using CEMRI if used as criteria to identify tumor foci (2).

There was a case of pseudoprogression correctly identified by DWI and FDG PET helping in differentiating thick nodular enhancement in that case is due to radiation necrosis with no recurrence. Though CEMRI and PWI techniques showed thick nodular enhancement with no abnormal perfusion.

In two cases simultaneous ASL was performed as part of the protocol. ASL, DWI, and PET volumes matched in both cases, and ASL which is a perfusion marker has good potential in tumor imaging. ASL is known to differentiate oligodendroglioma from astrocytoma irrespective of tumor grade and enhancement pattern and may aid in the pathological molecular typing of gliomas (3).

**Figure 3:**
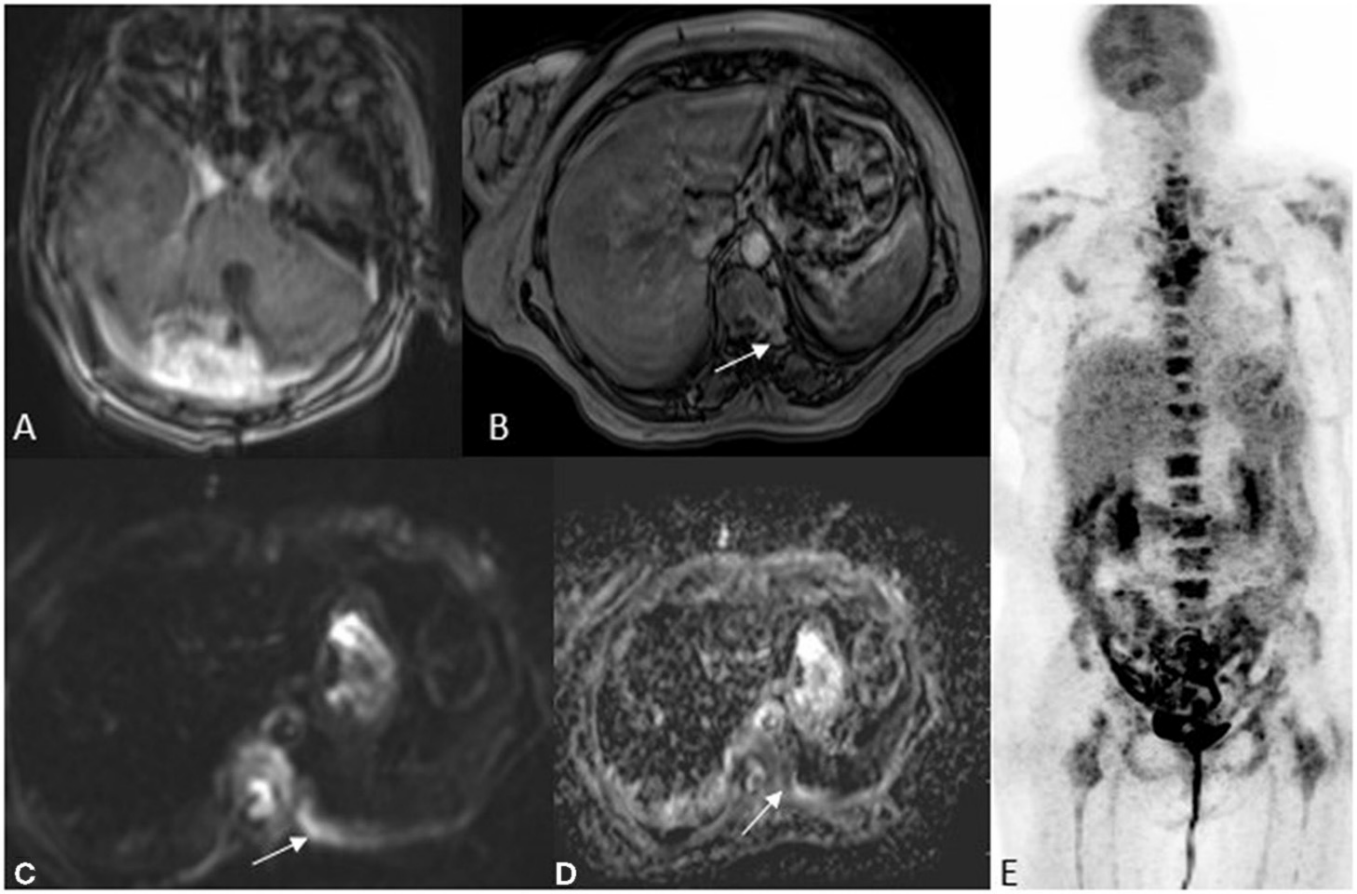
A 52 year old female with imbalance, MRI FDG PET axial brain images T1 post-contrast **(A)** image showed an enhancing lesion in the right cerebellum. Whole-body MRI with fast sequences at axial hepatic sections **(B)** VIBE fat sat post-contrast **(C)** DWI b800 **(D)** ADC and NAC PET **(E)** showing an area of metastases to the vertebral body as an area of restricted diffusion and post-contrast enhancement. HPE was suggestive of Pilocytic astrocytoma with metastasis.

Pilocytic astrocytoma with metastases is very rare. There was a histopathologically proven case and MR/PET corroborated this, proving metastatic spread. Whole-body DWI correlated well with FDG PET (Figure 3) (case 21).

There was a case of multiple intracranial lesions referred to as metastases. A whole-body MRI PET revealed it as a primary CNS tumor with metastases and both PET and DWI matched in the diagnosis (case 22).

### MRPET in Other CNS Tumor Histotypes

**Figure 4:**
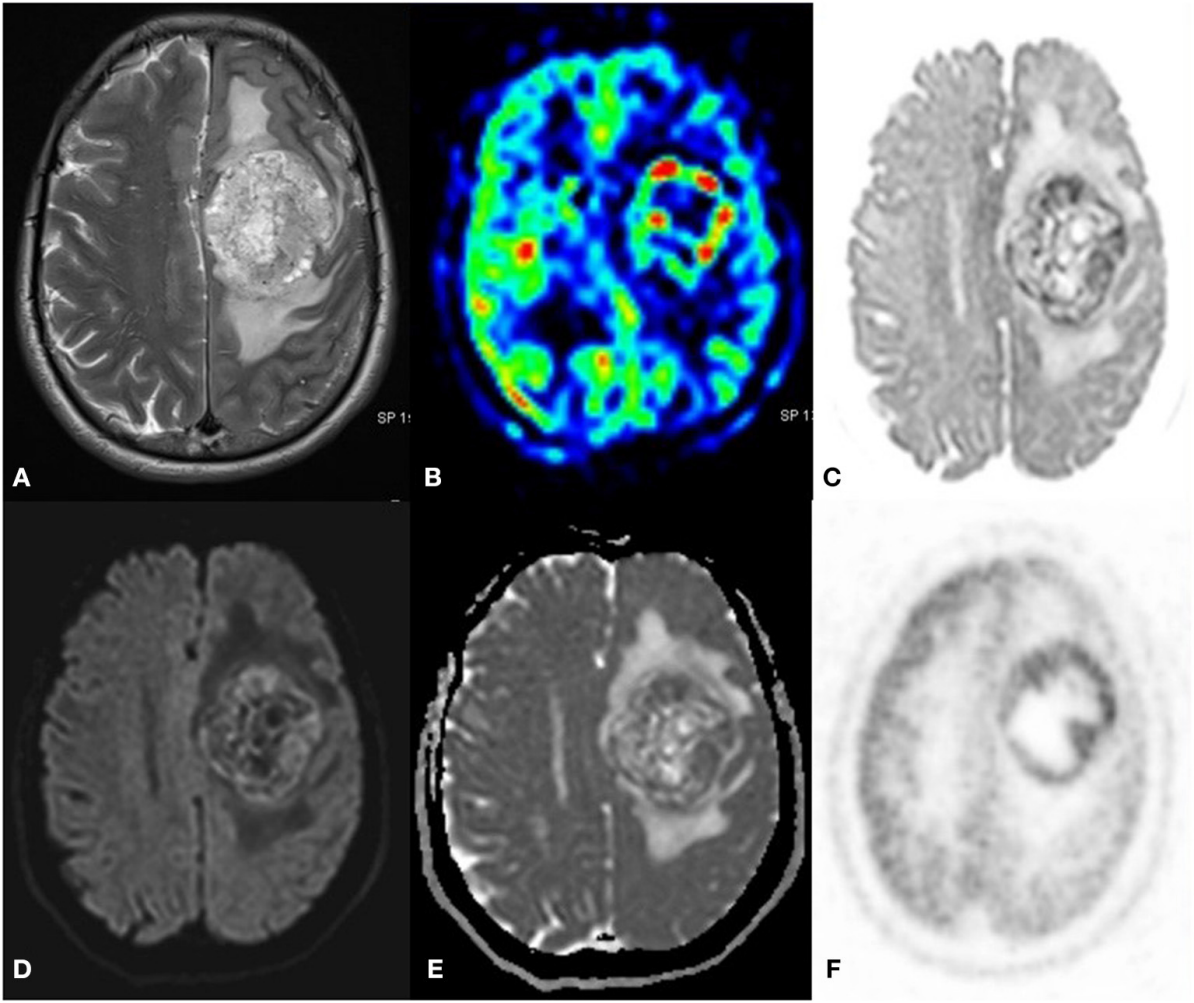
A 55 year old male with a progressive right-side weakness for one and a half month, MRI FDG PET axial images T2 **(A)** ASL **(B)** Inverted DWI **(C)** DWI b2000 **(D)** ADC **(E)** NAC PET **(F)** showed a well-defined lobulated T2 heterogeneous iso- hyperintense with increased perfusion on ASL. The lesion shows DWI restriction with hypermetabolism on PET. There is a match on the inverted DWI image and the NAC PET image. HPE is suggestive of Anaplastic Ependymoma.

Cases denote serial numbers in Table 2. We had a case of anaplastic Ependymoma (Figure 4), DWI, and FDG PET correlated well to confirm recurrence and rule out radiation necrosis though contrast enhancement patterns that appeared like radiation necrosis (Case 1).

There were 2 MR/PET cases of brain stem lesions diagnosed as demyelination on MR/PET and lymphoma in another, which on histopathology turned out to be T cell lymphoma and demyelination, respectively (Case 2, 3).

**Figure 5:**
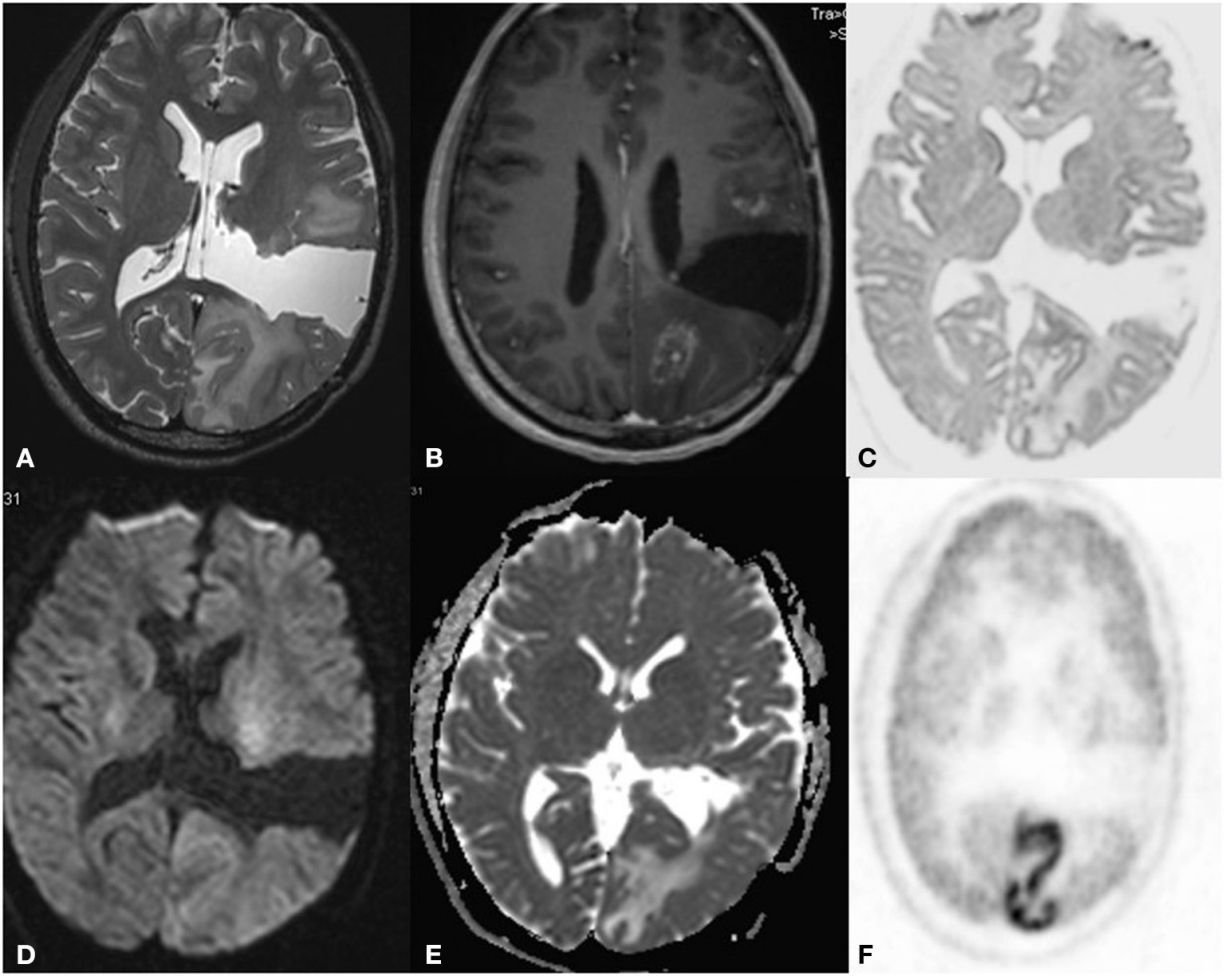
A 32 year old male postoperative, post-radiotherapy case of Anaplastic mixed oligoastrocytoma left thalamus, 3 year follow up MRI FDG PET axial images T2W **(A)** post-contrast T1 **(B)** Inverted DWI **(C)** DWI b2000 **(D)** ADC **(E)** NAC PET **(F)** show a postoperative cavity in the left parietal region. Anterior and posterior to this there is a heterogeneously enhancing lesion with elevated perfusion. The lesion shows DWI restriction with hypermetabolism on PET. This is suggestive of recurrence, but there is a large area of gyral enhancement due to SMART Syndrome. There is a false positive uptake on PET.

In another case, there was a large area of uptake on FDG mimicking recurrence but DWI, which showed only a small focus of restriction and due to DWI and FDG PET mismatch. PET with other tracers was done and DWI correlated well with F18 choline helped in planning the biopsy site. The patient had a sudden onset of transient weakness before FDG was injected and hence was diagnosed with SMART syndrome (stroke-like migraine attacks after radiation therapy) (case 4) (Figure 5).

There was a case of midline glioma H3K27 mutant, with extension into the thalamus and cerebellar peduncles DWI and FDG PET correlated well, post-radiotherapy follow up, DWI showed free diffusion suggesting resolution of the lesion (Case 5).

Another case with MRI T1 hyperintensity and CT hyperattenuating lesion in the right cerebellum and brachium pontis, suggested melanoma and it proved to be melanotic schwannoma. MR PET whole body was asked to rule out metastatic spread or any syndromic association (NF syndrome) before planning excision. There was no diffusion restriction. The enhancement pattern was confounded by the precontrast T1 hyperintensity. The FDG pet showed uptake as expected in schwannomas.

Whole-body MRI PET confirmed the SOL to be primary with no associated syndrome or metastatic spread, as the primary or secondary status could not be commented on histopathology. PET behavior was similar to any schwannoma and showed uptake. The T1 image identified it as a melanotic variant and DWI labeled it as low grade (case 7). This case highlights the importance of multimodality and whole-body imaging.

There was a case of lymphoma in the right external capsule, DWI- FDG correlated well, but there was remote hypermetabolism in bilateral frontal lobes mimicking multifocal lymphoma with no lesion noted on MRI (Case 9).

### Our Experience With Other Tracers

Tracers like F18 /C11 choline, C11 methionine, and Ammonia (NH3) PET were assessed.

**Figure 6:**
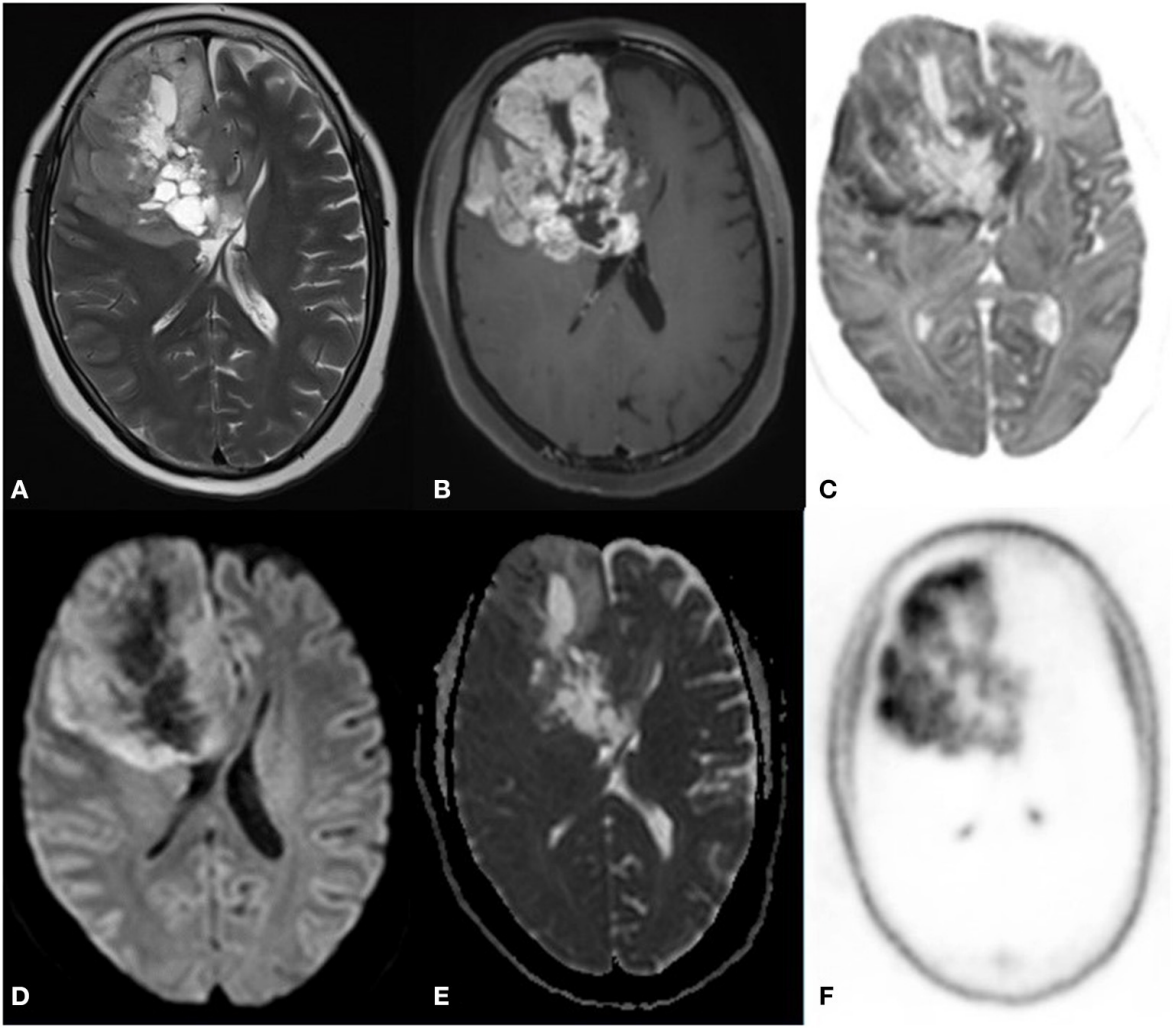
A 52 year old female with a headache for 1 year with recent onset of a seizure. MRI PET (F18 CHOLINE) axial images T2W **(A)** post-contrast TW **(B)** Inverted DWI **(C)** DWI b2000 **(D)** ADC **(E)** NAC PET **(F)** shows Ill-defined infiltrative heterogeneous lesion in the right frontal lobe with invasion to corpus callosum showing intense heterogeneous enhancement. The lesion shows DWI restriction with hypermetabolism on PET. There is a match on the inverted DWI image and the NAC PET image, which is suggestive of Glioblastoma.

DWI correlated well with FDG tracers like C11 methionine in cases of glioblastoma (Case 10–13) and F18 choline (Case 18–19) (Figure 6).

Methionine had better specific uptake in small foci of recurrence as compared to DWI, especially in low-grade glioma (Case 10).

**Figure 7:**
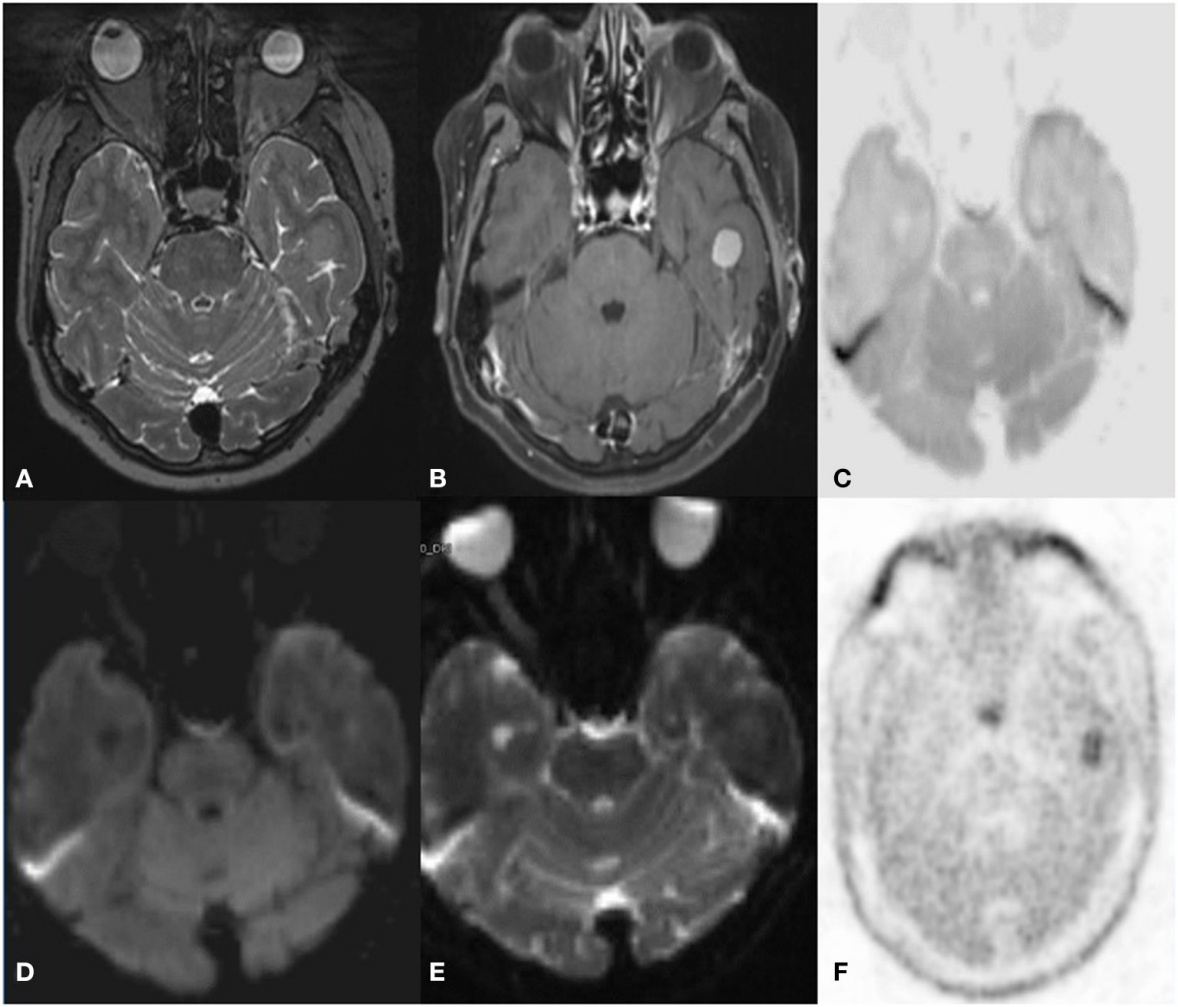
A 59 year old female with Glioma, incidentally detected a lesion in follow up MRI C-11 Methionine PET axial images T2W **(A)** post-contrast T1 **(B)** Inverted DWI **(C)** DWI b2000 **(D)** ADC **(E)** NAC PET **(F)** shows a broad-based T2 hypointense intensely enhancing in the left inferior temporal convexity. The lesion shows no DWI restriction with hypermetabolism on PET. S/O meningioma. False-positive uptake of meningioma by methionine.

There was a case of recurrence of glioma with an incidental meningioma (Figure 7), both recurrence and meningioma were picked by C11 methionine PET but DWI helped to differentiate the two. This case of meningioma showed no DWI restriction and had post-contrast brilliant enhancement. PET showed false-positive uptake irrespective of the grade of the tumor (Case 14).

There were cases of midline glioma in which both C11methionine PET and DWI correlated well. However, the intensity of uptake was more in methionine than DWI (Case 15).

Similar findings were noted in high-grade glioma recurrence in which DWI and methionine uptake was restricted to the tumor area, unlike FDG which has another non-specific uptake in the cortex, like in the case of SMART syndrome.

**Figure 8:**
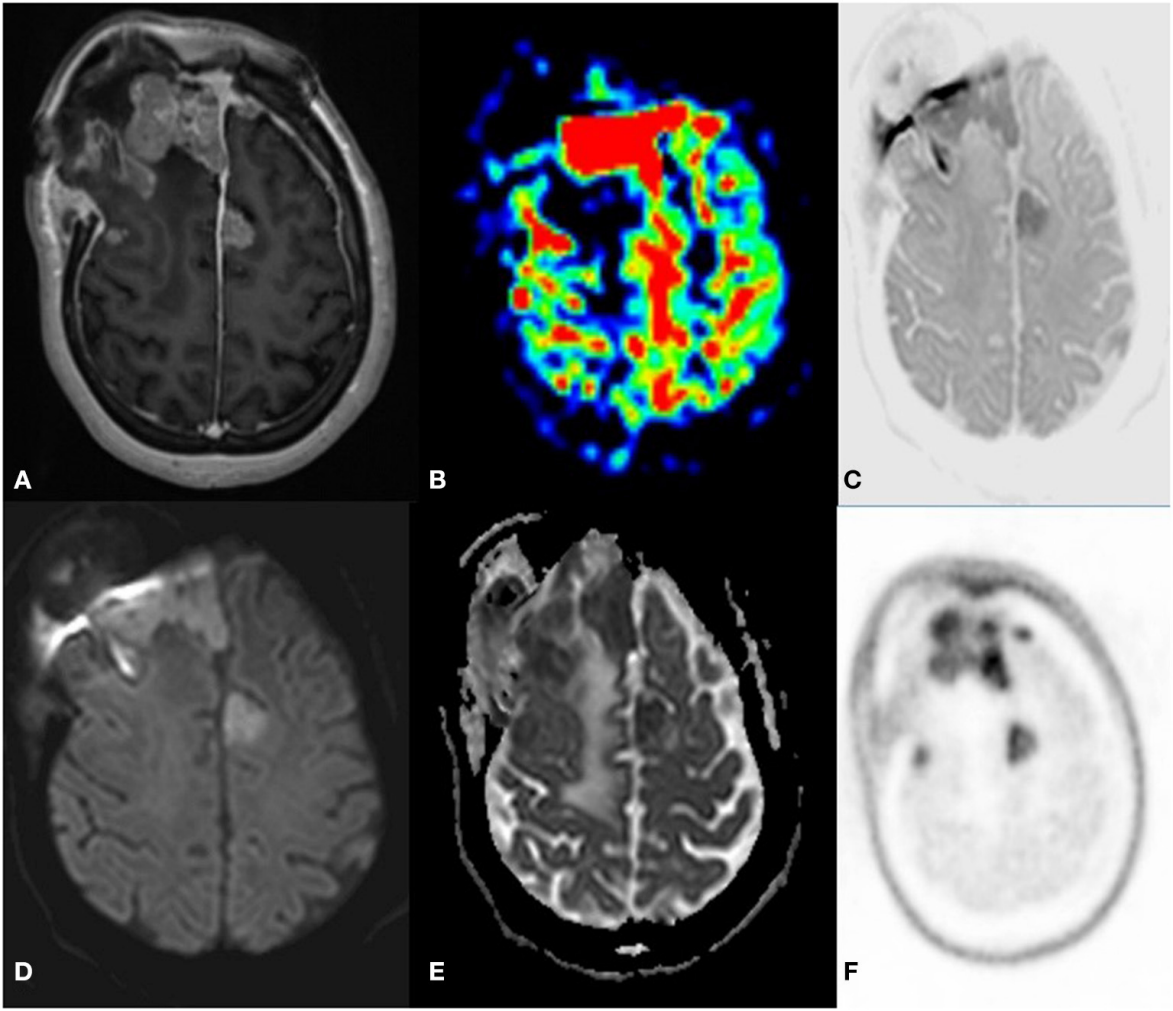
A 33 year old female postoperative, post-radiotherapy case of Embryonal tumor compatible (atypical teratoid rhabdoid tumor) 1 year follow up MRI C-11 Methionine PET axial images post-contrast T1 **(A)** ASL **(B)** Inverted DWI **(C)** DWI b2000 **(D)** ADC **(E)** NAC PET **(F)** showed Multiple enhancing dural based lesions are seen in bilateral frontal convexity and anterior falx with elevated perfusion. The lesion shows DWI restriction with hypermetabolism on PET. There is a match on the inverted DWI image and the NAC PET image, which is suggestive of recurrence.

In the case of the recurrent atypical teratoid rhabdoid tumor (Figure 8), DWI and C11methionine PET correlated well to confirm recurrence (Case 16). In another case of recurrent craniopharyngioma, DWI showed no restriction but uptake is seen in C11 methionine PET (Case 17).

**Figure 9:**
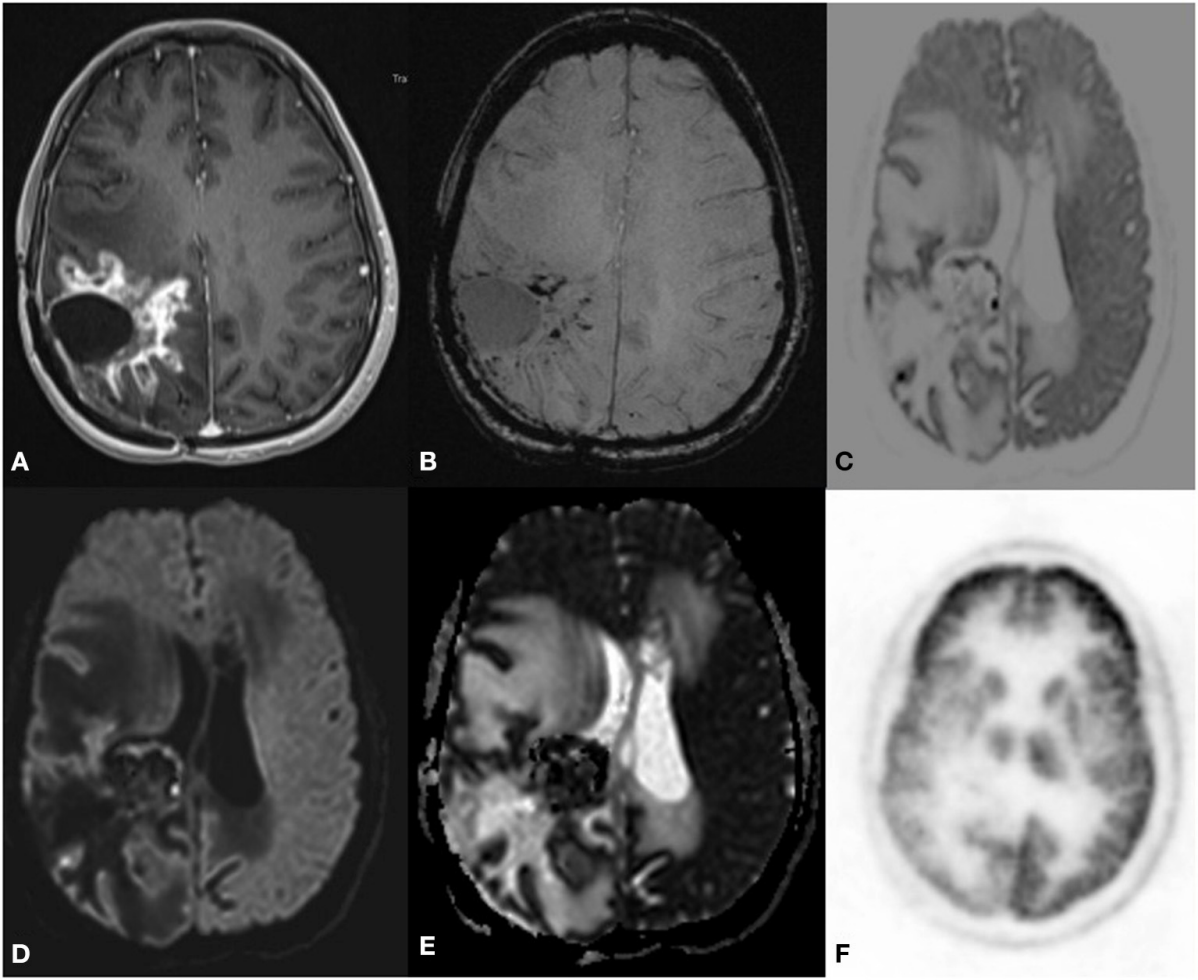
A 34 year old female postoperative case of anaplastic clear cell Ependymoma, 7 year follow up MRI F-18 choline PET axial images SWI **(B)** postcontrast T1 **(A)** Inverted DWI **(C)** DWI b2000 **(D)** ADC **(E)** NAC PET **(F)** showed a Swiss cheese and nodular enhancement lesion in the right parietal region. The lesion shows DWI restriction with hypermetabolism on PET. There is a match on the inverted DWI image and the NAC PET image, which is suggestive of recurrence.

We had the case of a clear cell variant of Ependymoma (Figure 9), evaluated with MR F18 choline PET. MRI DWI showed small foci of recurrence in the background of radiation necrosis. PET showed faint to no uptake as it was a clear cell variant and F18 choline has variable behavior in this subtype (4) (Case 20).

In all cases where there was DWI and PET, the correlation grayscale inverted b2000 image corresponded with the NAC image of PET, which has the potential to be an excellent biomarker in neuro-oncology workup.

### Discussion

This study evaluated the simultaneous acquisition of MR-DWI and PET data in the patients with suspected ICSOL, mainly focusing on gliomas and also other histotypes.

In lesion based sensitivity, the specificity of DWI was comparable with those of PET and in agreement with earlier published studies (5, 6).

In our study, we used the trace image as it nullifies T2 shine-through the effects of gliosis, and vasogenic edema, which is commonly associated with the tumor and postoperative and post-radiotherapy cases, and only tissue with increased cellularity is seen as bright on the trace image. A high b value (b2000) was generated to further decrease the background of the T2 effects and increase the contrast noise ratio. Synthetic high b value generation helps save time on the scanner and gives similar images, allowing retrospective analysis. We inverted this b2000 trace image to make it look like a PET AC corrected image. The ADC image does not have a good PET-like appearance and background parenchyma suppression is suboptimal to pick small foci.

This DWI-PET study suggests that DWI can be planned as a surrogate marker and saves time, which helps in surveillance imaging. Both DWI and PET have a resolution of 3–4 mm thick sections. In the case of tumor, DWI highlights cellularity and is immune from blood-brain barrier dysfunction and helpful in differentiating recurrence from enhancing radiation necrosis and in diagnosing non-enhancing recurrence (7). PWI and MRS have a good correlation but, since it is ROI-based, if the location of placement is wrong it can be misleading.

DWI and PET have a good one-on-one correlation (8). A combined DWI -PET increases true positive by nullifying the false positive and negative of both MRI and PET such as bleed, air/bone, the parenchyma interface artifacts of DWI, and the non-specific uptake of FDG (9).

Glioma is graded as 1–4 based on the cellularity/mitotic index. FDG PET has low sensitivity for grades 1 and 2, which is very similar to DWI (11). Excellent correlation was noted with FDG and DWI for tumor margin delineation in the background of radiation necrosis as compared to other MRI parameters (11). Tracers such as methionine and choline are better for low-grade tumors and recurrence of the low-grade tumor as compared to DWI–FDG PET in combination with conventional MRI for characterizing the lesion further into a meningioma or a glioma etc.

The intensity of uptake and DWI grayscale matches with FDG but not with other tracers. Tumor volume was better delineated with DWI and methionine as compared to FDG and GHA wherein lesions with increased SUV often had exaggerated volumes and a spill-over effect. The FDG intensity reflects the metabolic behavior and can help plan the intensity of radiotherapy and prognosticate whereas MRI can help define borders (12).

This paper highlights the role of a high b value DWI weighted image trace image with grayscale inversion, which is excellent for achieving a PET-like image. Since a close correlation between DWI and FDG has been established, developing high b value DWI as a biomarker can help in treatment response assessment in close intervals without risk of contrast, radiation, and cost.

Our experience of DWI–PET with other histotypes of brain tumor and tracers highlighted the need to know false positive and negative rates associated with FDG and other tracers and interpret PET along with MRI parameters (13) such as DWI, ASL, PWI, MRS, SWI and CEMRI for further characterization and diagnosis. False-positive uptake in meningioma (9) was noted with free diffusion and brilliant enhancement. This type of MR correlation is very important in syndromes such as Neurofibromatosis wherein both glioma and meningioma can coexist and identify malignant transformation. Similarly, brain tuberculoma can show raised FDG uptake and can mimic tumors with high SUV (14) but MRI DWI can show free diffusion and aids in differentiating them (15).

Simultaneous MR/PET of the brain and whole body helps confirm whether a lesion is a primary CNS lesion rather than metastases/systemic lymphoma. Overall hybrid imaging has many advantages such as increasing the diagnostic accuracy to demarcate viable tumor margin, planning therapy, and patient management and treatment response (16). Knowledge of the advantages and pitfalls of each modality is required. CT MRI provides excellent cross-sectional imaging with MRI having a resolution of up to 1 mm as compared to PET, which has a resolution of 4 mm. Structural MRI has excellent spatial resolution and is well-suited to differentiate tumors from edema, hemorrhage other features such as raised ICP. Advanced MRI such as ASL, DWI, PWI, SWI, and MRS also provide blood flow cellularity and metabolic information (10).

PET tracers give information on subcellular processes based on the radiopharmaceutical used such as glucose/amino acid metabolism and so on. Though tumor biology is associated with tracers to map increased glucose consumption, increased expression of amino acid transporters, increased proliferation rate, increased membrane biosynthesis, increased perfusion, and hypoxia are available and a commonly used tracer is glucose consumption imaging. FDG reflects increased tumor glucose metabolism, glycolysis via the GLUT receptors, and apoptosis rate /mitotic index in tumors. Some low-grade tumors with increased expression of GLUT receptors such as pilocytic astrocytoma and neuroma etc. can show high uptake of FDG. In these cases, MRI DWI can help differentiate benign tumors from high-grade lesions (17).

Amino acid PET imaging such as ammonia, methionine, FET, FLT, FDOPA deals with part of tumor biology related to histopathology with the Ki-67 index, proliferating cell nuclear antigen, and microvessel density (18).

A knowledge of biological distribution of different tracers in brain is required before interpretation for example ammonia is transported by process of diffusion and others are carrier mediated and dependent on BBB damage. Areas which lack BBB such as choroid plexus and pituitary gland can show uptake (F-thymidine and F choline) (19). Choice of tracers for brain tumor in these location should be planned accordingly. FLT may have lower specificity for low grade tumors as compared to methionine PET.

Methionine has a better background suppression as compared to choline and thymidine and is preferred in tumor imaging. Normal distribution of this tracer should be known and non tumor conditions such as demyelination or abscess can also show methionine uptake (20).

F DOPA is another tracer but can be taken up by other sol such as meningioma etc. Overall since FDG, methionine and FDOPA uptake is affected by body metabolism and knowledge of patient preparation is required such as controlled sugars, seizure free, low protein diet, off dopaminergic drugs for the above tracers (21).

In our series we don't have experience with FET, FLT or FDOPA for tumour and its correlation with DWI or MRPWI. Hypoxia imaging with FMISO helps demarcate tumor margins and angiogenesis. Perfusion imaging with radio water reflects angiogenesis that correlates with VEGF and antigen ki6 on histopathology which make tumor more aggressive and resistant to radiotherapy (22). Molecular imaging with hybrid technology expands the scope of tracers and imaging *in vivo*. Newer chemistry derivatives can be used either with Gadolinium particles, Nano particles for MRI or radiotracers for PET or fluorescein for optical imaging. It is an exciting field for *in vivo* imaging (16).

This paper has some limitations. First, the limited sample size and study design involved visual interpretation rather than voxel-wise correlation by fusing DWI and PET images because of quick analysis, reproducibility, and non-time consumption. Rather than highlighting the differences between DWI and PET, this paper says that both are complementary. The difference between the image and the representative histopathological specimen limited the proof of concept.

The preliminary results of this study need to be confirmed in a larger patient population. Further studies are planned to extend parameter inclusion and quantification. The main strength of this paper is that this study involves a broader spectrum of CNS ICSOL imaged with multiple types of PET tracers and all cases have been followed up to establish the diagnosis.

## Conclusion

The role of imaging is not about characterizing the lesion but trying to extract virtual histopathology-like features *in vivo* and, additionally, keep pace with newer modes of tumor treatment regimens that target different pathways of a tumor. This approach has led to the development of multimodality advanced hybrid imaging. Although DWI and FDG-PET reflect different tissue properties, there may very well be an association between the measures of both methods, most probably because of increased cellularity and the glucose metabolism of FDG-avid CNS lesions. Presently PET is an adjunct to MRI in neurooncology. DWI helps pick the lesion, calculate tumor volume, and predict appropriate early post-treatment tumor response. MRI is a good tool when multi-time point imaging is required for the diagnosis of a tumor, for planning therapy, post-treatment response, and further surveillance. DWI acts as a surrogate to FDG PET and has promising potentials for clinical translation.

## Data Availability Statement

The original contributions presented in the study are included in the article/supplementary material, further inquiries can be directed to the corresponding author/s.

## Ethics Statement

The studies involving human participants were reviewed and approved by Ethics Committee Board, NIMHANS. Written informed consent for participation was not required for this study in accordance with the national legislation and the institutional requirements.

## Author Contributions

SM contributed to the original concept, image analysis, the manuscript draft, and clinical MRI reporting. SV and SJ undertook data collection and manuscript drafting. AK contributed to manuscript overview and inputs. PK contributed for production of PET tracers at the institute cyclotron. All authors contributed to the article and approved the submitted version.

## Conflict of Interest

The authors declare that the research was conducted in the absence of any commercial or financial relationships that could be construed as a potential conflict of interest.

## Publisher's Note

All claims expressed in this article are solely those of the authors and do not necessarily represent those of their affiliated organizations, or those of the publisher, the editors and the reviewers. Any product that may be evaluated in this article, or claim that may be made by its manufacturer, is not guaranteed or endorsed by the publisher.
